# Outcomes and Complications of Bipolar vs. Monopolar Energy for Transurethral Resection of Bladder Tumors: A Systematic Review and Meta-Analysis of Randomized Controlled Trials

**DOI:** 10.3389/fsurg.2021.583806

**Published:** 2021-06-02

**Authors:** Xin Mao, Zhongbao Zhou, Yuanshan Cui, Yong Zhang, Mingshan Yang

**Affiliations:** ^1^Department of Urology, Affiliated Hospital of Qingdao University, Qingdao, China; ^2^Department of Urology, Beijing TianTan Hospital, Capital Medical University, Beijing, China; ^3^Department of Urology, The Affiliated Yantai Yuhuangding Hospital of Qingdao University, Yantai, China; ^4^Department of Urology, Shandong Cancer Hospital and Institute, Shandong First Medical University and Shandong Academy of Medical Sciences, Jinan, China

**Keywords:** monopolar and bipolar, transurethral resection, bladder cancer, randomized controlled trials, systematic review and meta-analysis, complications

## Abstract

**Background:** Bipolar and monopolar transurethral resections have a stable status for non-muscle invasive bladder cancer (NMIBC). We conducted a meta-analysis to analyze the outcomes and complications of bipolar vs. monopolar energy for transurethral resection of bladder tumors (TURB).

**Methods:** The Preferred Reporting Items for Systematic Reviews and Meta-analyses was followed. Based on the Population, Intervention, Comparator, Outcomes, and Study Designs (PICOS) strategy, randomized controlled trials were searched in MEDLINE, EMBASE, and the Cochrane Controlled Trials Register. The reference lists of the associated articles were also retrieved. The data were calculated by Rev Man v5.3.0.

**Results:** Eleven publications containing an amount of 2, 099 patients were involved in the study. Two groups did not show a significant difference in the mean age and the number of bladder tumors. The results showed that m-TURB had a greater decrease in postoperative hemoglobin level [mean difference (MD) −0.26, 95% confidence interval (CI) −0.48 to −0.04, *P* = 0.02] and sodium level (MD −0.36, 95% CI −0.62 to −0.10, *P* = 0.007) compared with b-TURB. B-TURB spent relatively little in hospitalization time (MD −0.52, 95% CI −0.88 to −0.15, *P* = 0.005) than m-TURB with the exception of operation time (*P* = 0.47) and catheterization time (*P* = 0.19). B-TURB did not show a significant difference in the incidence rate of obturator reflex (*P* = 0.10), bladder perforation (*P* = 0.32), postoperative blood transfusion (*P* = 0.28), and clot retention (*P* = 0.21) compared with the b-TURB group. Besides, there were no significant difference in terms of muscle tissue sampling (*P* = 0.43), recurrence-free survival at 6 months (*P* = 0.68) and 12 months (*P* = 0.78).

**Conclusions:** B-TURB was more effective than m-TURB in minimizing intraoperative or postoperative bleeding with the smaller loss of hemoglobin and the shorter hospitalization time for patients with NMIBC.

## Introduction

Bladder cancer is one of the most common malignancies in the United States, and its incidence rate is about 80,470 new cases and 17,670 deaths in 2019 (1, 2). In new cases, non-muscle invasive bladder cancer (NMIBC) accounts for ~80%, and urothelial carcinoma is the main type of histologic classification (3, 4).

Transurethral resection (TUR) is the basis of staging and treatment of bladder tumors (5). Transurethral resection of bladder tumors (TURB) aims to achieve a definitive diagnosis and remove visible pathological tissue including muscle tissue (6). Monopolar activated current, as a source of energy for the cutting loop, is the gold standard for the treatment of NMIBC, which is simultaneously correlated with some adverse events including blood loss and disorder of electrolyte balance (7). Recently, bipolar resection with the use of physiological saline solution has proven to be a beneficial choice in the prevention of possible complications (8–10). However, there were few sufficient evidence-based medical studies focusing on analyzing the advantages and disadvantages of the two technologies for bladder tumors.

We conducted a meta-analysis of randomized controlled trial (RCTs) to evaluate the outcomes and complications of bipolar vs. monopolar TURB.

## Materials and Methods

### Study Protocol

This systematic review was implemented by following the Preferred Reporting Items for Systematic Reviews and Meta-Analyses (PRISMA) checklist (11). Only RCTs were included in the study. Observational studies, editorials, commentaries, and review articles were excluded. Abstracts of conferences were also excluded. If a group of patients was included in two or more studies, each of the studies may have been analyzed in the present study.

### Information Sources and Literature Search

Based on databases including MEDLINE (1996 to May 2020), EMBASE (1999 to May 2020), and the Cochrane Controlled Trials Register, two reviewers did a comprehensive retrieval to analyze the outcomes and complications of b-TURB vs. m-TURB. The following search terms were used: “bipolar, monopolar, TURB, and NMIBC.” The study only included published literature with restriction on English language. If necessary, authors were contacted to provide more accurate data for their research. Meanwhile, reviewers also searched for published systematic reviews and other key references. Two reviewers screened independently titles and abstracts to identify studies that met the inclusion criteria, and if there were any objections, it was referred to the third person for examination. When abstracts were insufficient to determine whether the study met the inclusion criteria, full text would be required.

### Eligibility Criteria

**Figure 1:**
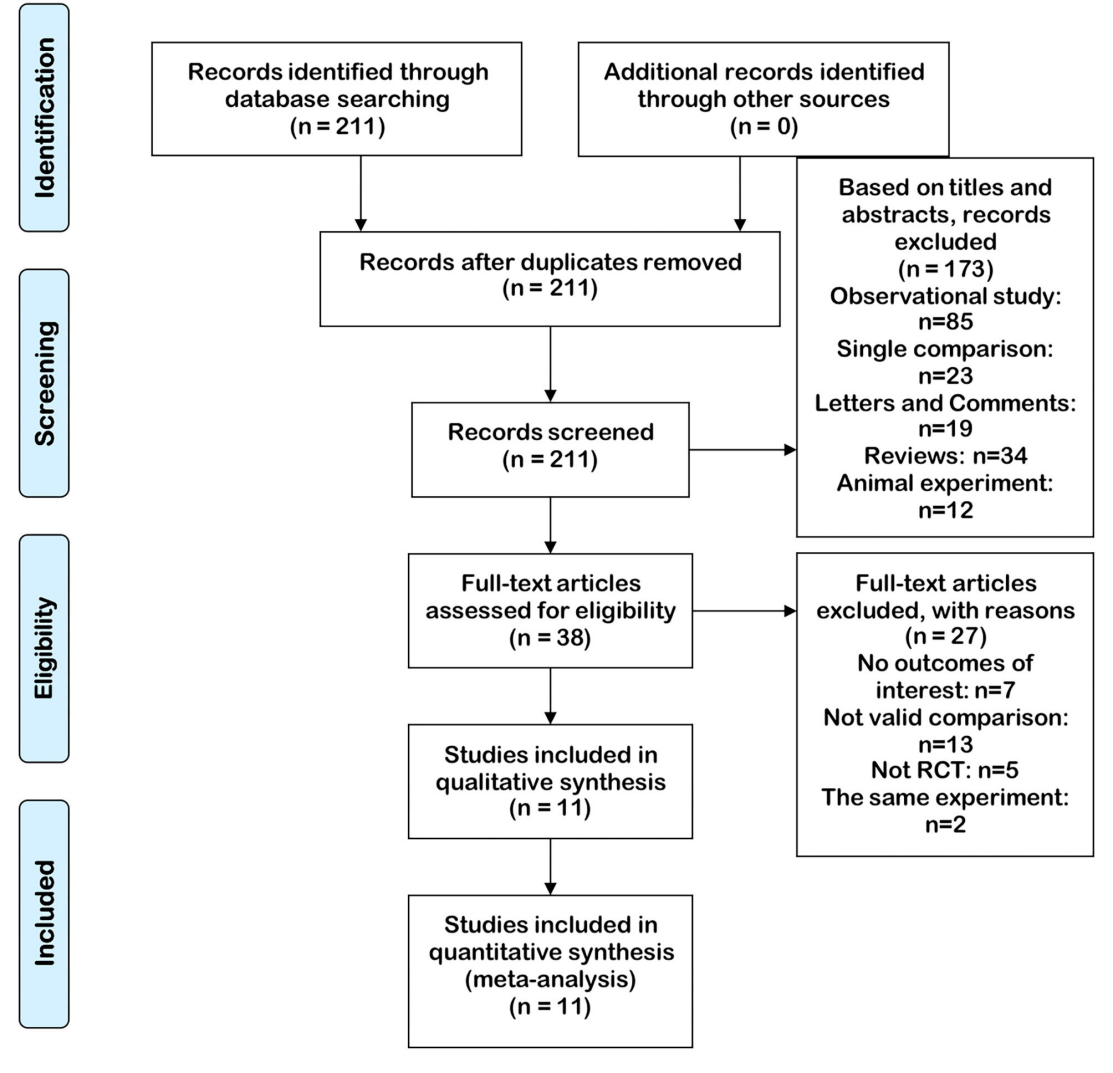
Flowchart of the study selection process. RCT, randomized controlled trials.

(1) B-TURB vs. m-TURB was involved. (2) Full-text could be acquired. (3) The data provided by the article were valid and valuable, mainly involving the number of cases and valuable results of each indicator. (4) The method of article was RCT. The search strategy according to the focused PICOS question is presented in Table 1. The PRISMA diagram of selection is shown in Figure 1.

**Table 1:** Criteria for considering studies for the review based on the Population, Intervention, Comparator, Outcomes, and Study Designs (PICOS) Structure.

	**Population**	**Intervention**	**Comparator**	**Outcomes**	**Study designs**
Inclusion criteria	Patients with at least one bladder tumor for overt or suspected bladder cancers on radiological imaging and/or cystoscopy	Bipolar technology	Monopolar technology	Hemoglobin level, hematocrit level, sodium level, operation time, catheterization time, hospitalization time, obturator reflex, bladder perforation, postoperative blood transfusion, clot retention, muscle tissue sampling, recurrence-free survival at 6- and 12-months, TUR syndrome, postoperative severe cautery artifact	RCT
Exclusion criteria	Acute urinary tract infection, absence of urethelial cancer on pathology report after TURB, presence of muscle invasive bladder cancer after endoscopic resection, etc.	Other therapy	Other therapy	Qualitative outcomes such as patient feelings; Inadequate indicators	Observational study, letters, comments, reviews, and animal experiment

*TURB, transurethral resection of bladder tumors; RCT, randomized controlled trial; TUR, transurethral resection*.

### Quality Assessment Methods

Each study was classified by the Jadad scale (12). Studies were graded in line with the principles, which are derived from the *Cochrane Handbook for Systematic Reviews of Interventions v5.30* (13). Each RCT was allotted according to the following quality classification standards: (+) low potential of bias, (?) secondary probability of bias, and (–) high possibility of bias. All authors participated in the assessment of each RCT, and eventually, everyone agreed with the results. All reviewers independently assessed whether the study fitted into the criteria. Any discrepancies were recorded, discussed, and settled in a negotiated manner.

### Data Extraction

Based on predetermined criteria, three reviewers independently extracted the following data from each study: (A) published time; (B) first author's name; (C) country of study; (D) the type of trial; (E) technique received; (F) number of participants; (G) mean age; (H) tumor number (single/multiple); and (I) hemoglobin level, hematocrit level, sodium level, operation time, catheterization time, hospitalization time, obturator reflex, bladder perforation, postoperative blood transfusion, clot retention, muscle tissue sampling, and recurrence-free survival at 6 and 12 months. Because these indicators had a remarkable impact on patient, they were regarded as an important aspect. No ethical approval was required for our study.

The main primary outcomes were operative time, catheterization time, and hospitalization time. The secondary outcomes were postoperative complications, tumor muscle tissue sampling, and recurrence-free survival at 6 and 12 months.

### Statistical Analyses

Reman version 5.3.0 (Cochrane Collaboration, Oxford, UK) (13) was used in the analysis of data. Mean difference (MD) was applied to analyze continuous data, and the odds ratio (OR) was calculated for dichotomous results with the corresponding 95% confidence interval (CI) (14). The results of analysis showed that if the *P* > 0.05 for the *I*^2^ statistic, the study was considered to be homogeneous, and the fixed-effects model was used for the analysis. When heterogeneity is high, sensitivity analysis and subgroup analysis would be used to analyze the source of heterogeneity if necessary, which may understand the potential confounders that might be significantly associated with the outcomes of interest (i.e., tumor features, patient features, etc.). Otherwise, a random-effects model would be used in the study when the results showed *p* < 0.05. For the main evidence from RCTs, we rated our confidence in the estimates of effect for the outcome as strength of evidence (SOE) as high, moderate, low, or insufficient (15).

## Results

### Study Selection Process, Search Results, and Characteristics of the Trials

Our search found 211 articles by retrieving three databases. In screening abstracts and titles, 173 articles were excluded. For the remaining 38 articles, 25 articles were excluded due to lack of available data, and two articles were excluded because of the same trial. Finally, 11 articles containing 11 RCTs (16–26) were included to evaluate the outcomes and complications of b-TURB vs. m-TURB. The details of 11 articles are listed in Table 2. Two groups did not show a significant difference in the mean age and the number of bladder tumors. Patients with NMIBC included in each study showed a similar evaluation index.

**Table 2:** The details of individual study.

**Study**	**Country**	**Study design**	**Technique**		**Sample size**		^*^Mean age (years) ± SD		^*^Tumor (Single/Multiple)		**Main inclusion criteria**
			**Experimental**	**Control**	**Experimental**	**Control**	**Experimental**	**Control**	**Experimental**	**Control**	
Geavlete et al. (16)	Romania	RCT	Bipolar technology	Monopolar technology	60	60	62.5 (36–86)	61.7 (34-85)	23/37	26/34	Patients with at least one bladder tumor larger than 3 cm
Del Rosso et al. (17)	Italy	RCT	Bipolar technology	Monopolar technology	67	65	64.9 (56–77)	66.3 (54-81)	56/11	54/11	Patients with newly diagnosed NMIBC by using ultrasonography, contrast-enhanced computed tomography and cystoscopy
Venkatramani et al. (18)	India	RCT	Bipolar technology	Monopolar technology	72	75	55.2 ± 12.4	55.5 ± 12.5	N/A		Patients who undergoing TURBT for suspected bladder tumors
Teoh et al. (19)	Hong Kong, China	RCT	Bipolar technology	Monopolar technology	75	79	72.9 ± 12.1	73.6 ± 11.1	45/30	45/34	Patients who have a bladder tumor (either primary or recurrent), and who planned for TURBT
Thirugnanasambandam and Ramanathan (20)	India	RCT	Bipolar technology	Monopolar technology	50	50	56.5 ± 10.4 (32–80)	58.2 ± 8.45 (40–76)	45/5	46/4	Patients diagnosed to have bladder tumor by using ultrasonography, contrast-enhanced computed tomography and cystoscopy
Balci et al. (21)	Turkey	RCT	Bipolar technology	Monopolar technology	119	117	59.5 ± 13.8	62.3 ± 12.9	55/64	69/48	Patients were diagnosed preoperatively with≥1 apparently NMIBT >3 cm in diameter
Hashad et al. (22)	Egypt	RCT	Bipolar technology	Monopolar technology	100	100	59.37 ± 7.14 (46–81)	59.37 ± 7.14 (45–80)	70/30	68/32	Patients presenting with bladder tumors of >3 cm in maximum diameter, as measured by ultrasonography
Bolat et al. (23)	Turkey	RCT	Bipolar technology	Monopolar technology	48	42	73.71 ± 8.15	71.36 ± 7.49	N/A		Patients who underwent TURBT for overt or suspected bladder cancers on radiological imagings and/or cystoscopy
Gramann et al. (24)	Switzerland	RCT	Bipolar technology	Monopolar technology	23	21	74.4 (58–91)	69.4 (51–82)	N/A		Patients scheduled for elective TURB with a newly diagnosed or recurrent bladder tumor
Liem et al. (25)	Multicenter study	RCT	Bipolar technology	Monopolar technology	406	310	66.9 ± 11.8	66.6 ± 11.9	N/A		Patients had primary NMIBC treated with mTURB or bTURB
Murugavaithianathan et al. (26)	India	RCT	Bipolar technology	Monopolar technology	80	80	57.93 ± 17.93	58.50 ± 12.02	65/15	70/10	Patients with bladder tumor undergoin TURBT under regional anesthesia

* *No significant difference between experimental group and control group (P = 0.45 and 0.14, respectively)*.

*RCT, randomized controlled trial; SD, standard deviation; NMIBC, non-muscle invasive bladder cancer; TURBT, transurethral resection of the bladder tumor; mTURB, monopolar TURB; bTURB, bipolar TURB; N/A, not available*.

### Risk of Bias

**Figure 2:**
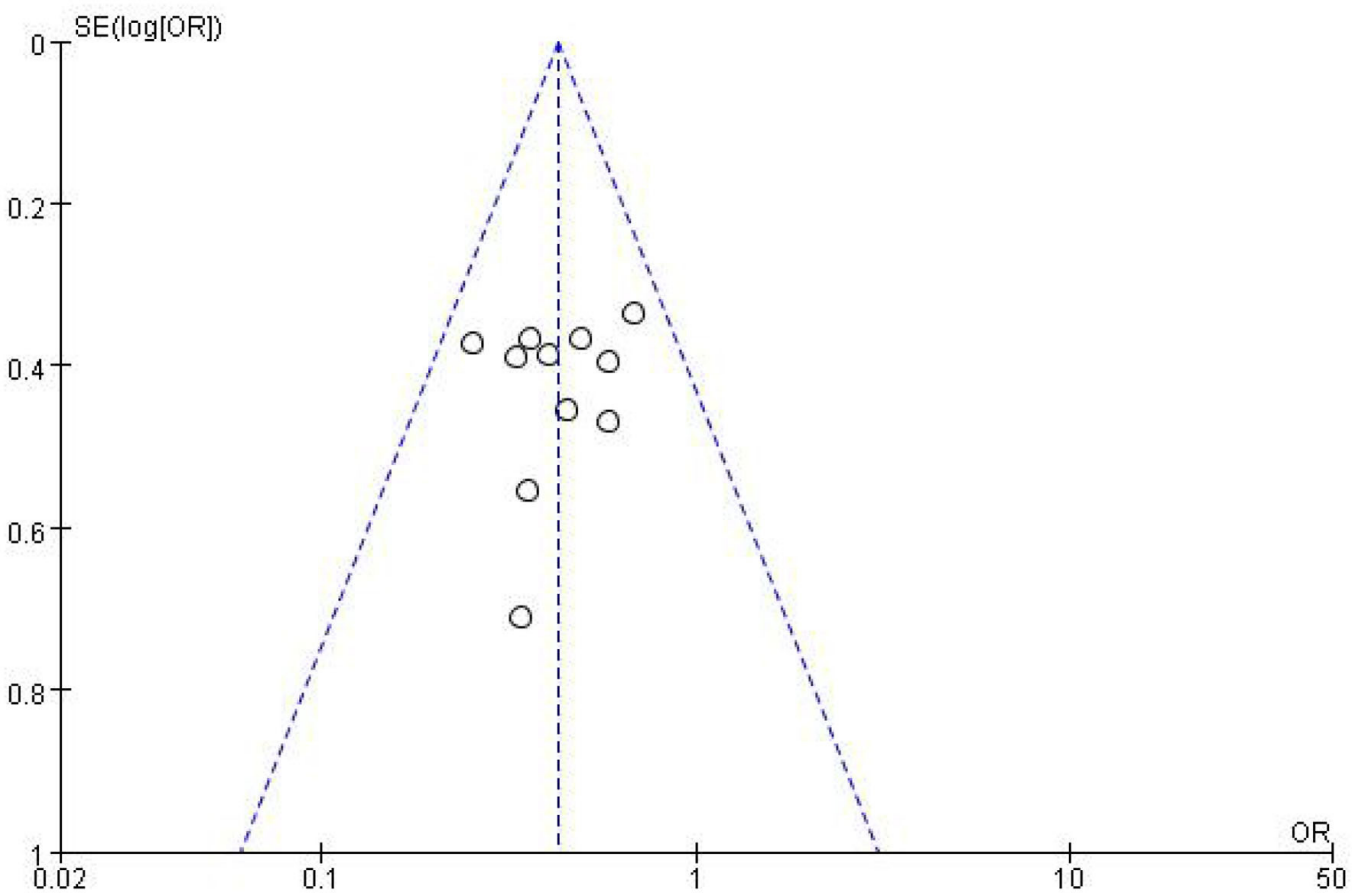
Funnel plot of studies included in the meta-analysis. OR, odds ratio; SE, standard error.

**Figure 3:**
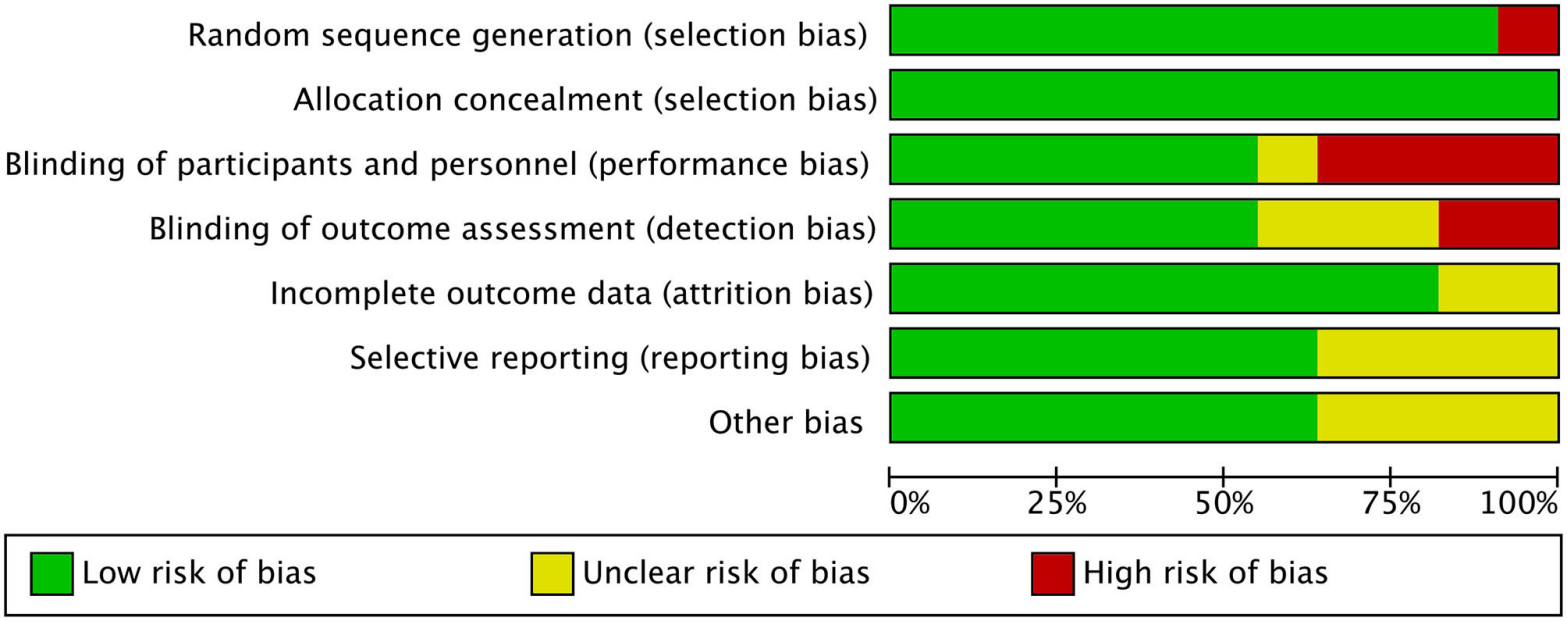
Risk of bias summary.

**Figure 4:**
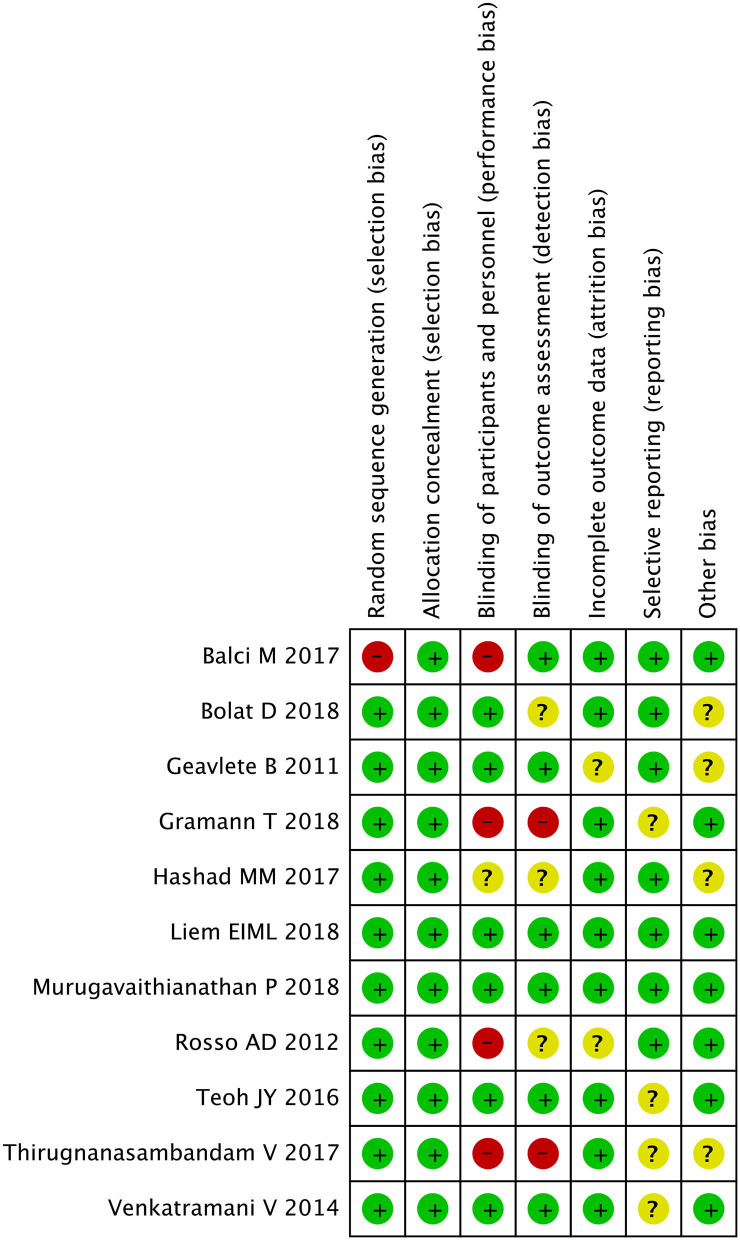
Risk of bias graph.

All studies included in the meta-analysis were RCT. The plot showed that 11 circles were contained in the large triangle, and no evidence of bias was found (Figure 2). The risk of bias summary and graph are shown in Figures 3, 4.

### Primary Outcomes

#### Hemoglobin Level

**Figure 5:**
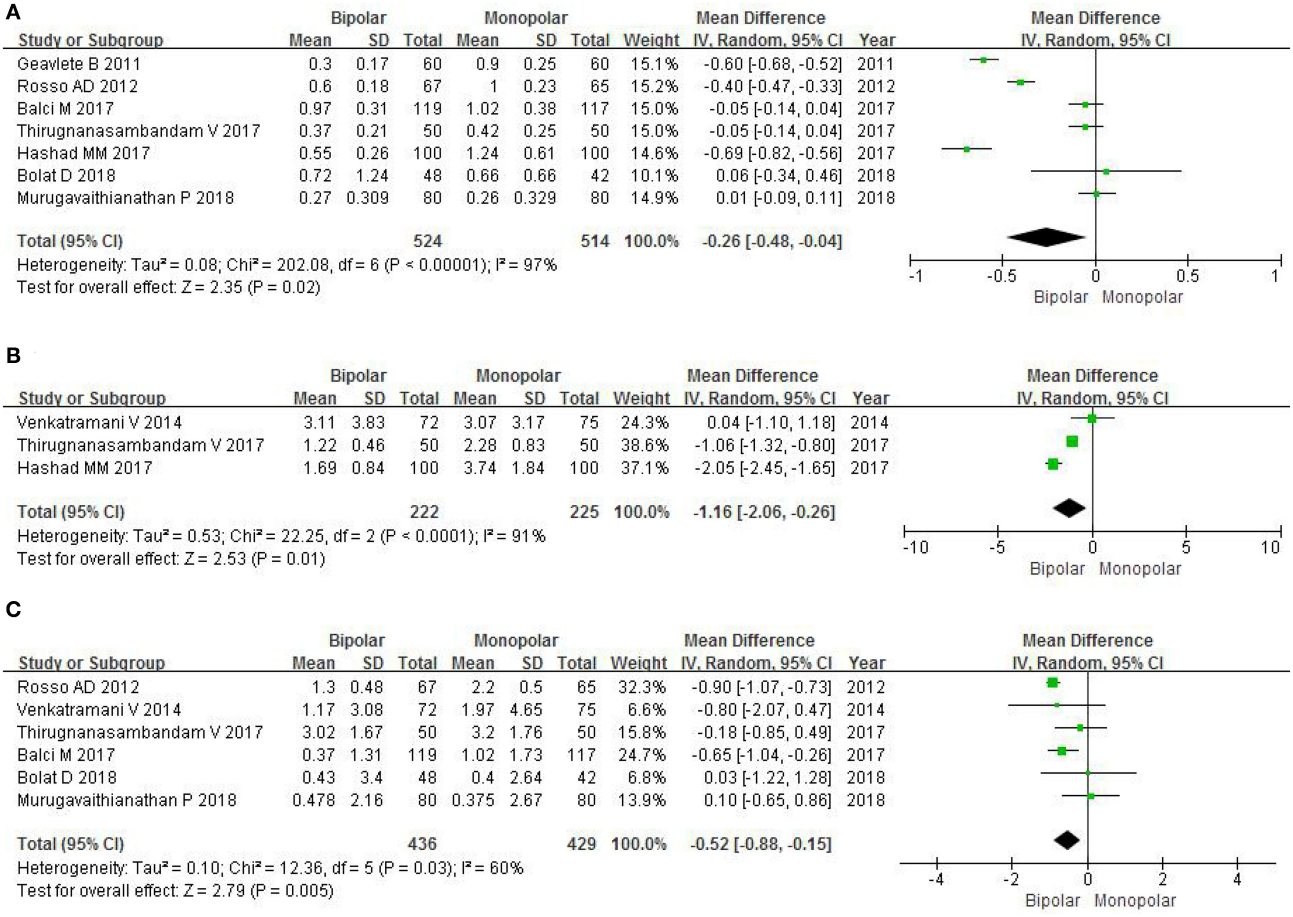
Forest plots showing changes in **(A)** hemoglobin level; **(B)** hematocrit level; **(C)** sodium level. SD, standard deviation; IV, inverse variance; CI, confidence interval; df, degrees of freedom.

Seven RCTs gathering a total of 1,038 patients contributed to access a decrease in hemoglobin level. The forest plot demonstrated that m-TURB had a greater decrease in postoperative hemoglobin level (MD −0.26, 95% CI −0.48 to −0.04, *P* = 0.02; Figure 5A) compared with b-TURB. This result suggested that b-TURB was more effective than m-TURB in terms of minimizing bleeding during the procedure.

#### Hematocrit Level

To evaluate the decrease in hematocrit level, three RCTs had a sample of 447 patients. The random effect estimate of MD was −1.16, and the 95% CI was −2.06 to −0.26 (*P* = 0.01; Figure 5B). This result indicated that b-TURB was more effective in reducing the amount of bleeding compared with m-TURB.

#### Sodium Level

Six RCTs with an amount of 865 patients included data on the change of sodium level. The forest plots showed that m-TURB had a significant decrease in postoperative sodium level compared with b-TURB (MD −0.36, 95% CI −0.62 to −0.10, *P* = 0.007; Figure 5C).

#### Operation Time

**Figure 6:**
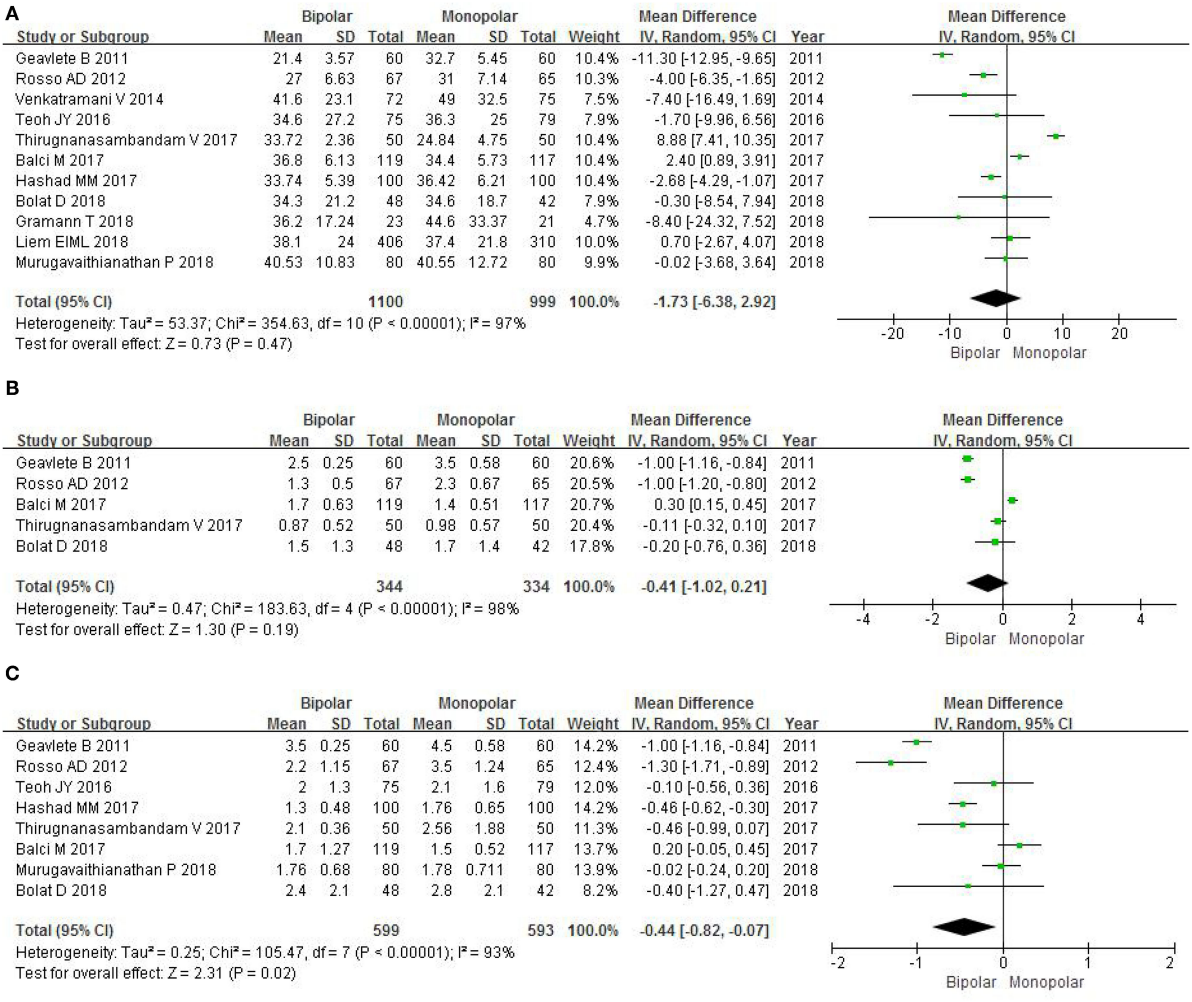
Forest plots showing length in **(A)** operation time; **(B)** catheterization time; **(C)** hospitalization time. SD, standard deviation; IV, inverse variance; CI, confidence interval; df, degrees of freedom.

Eleven RCTs enrolling 2,099 patients contained the data of operation time. The forest plots showed an MD of −1.73 and 95% CI of −6.38 to 2.92 (*P* = 0.47; Figure 6A). We found no statistical significance between b-TURB and m-TURB in the duration of the surgery.

#### Catheterization Time

Five RCTs gathering 678 patients included the data of catheterization time. The forest plots did not show a marked difference between b-TURB and m-TURB in the duration of the catheterization (MD −0.41, 95% CI −1.02 to 0.21, *P* = 0.19; Figure 6B).

#### Hospitalization Time

Eight RCTs gathering 1,192 patients included the data of hospitalization time. The forest plots showed that b-TURB spent less time in the hospital compared with m-TURB (MD −0.44, 95% CI −0.82 to −0.07, *P* = 0.02; Figure 6C).

### Secondary Outcomes

#### Obturator Reflex

**Figure 7:**
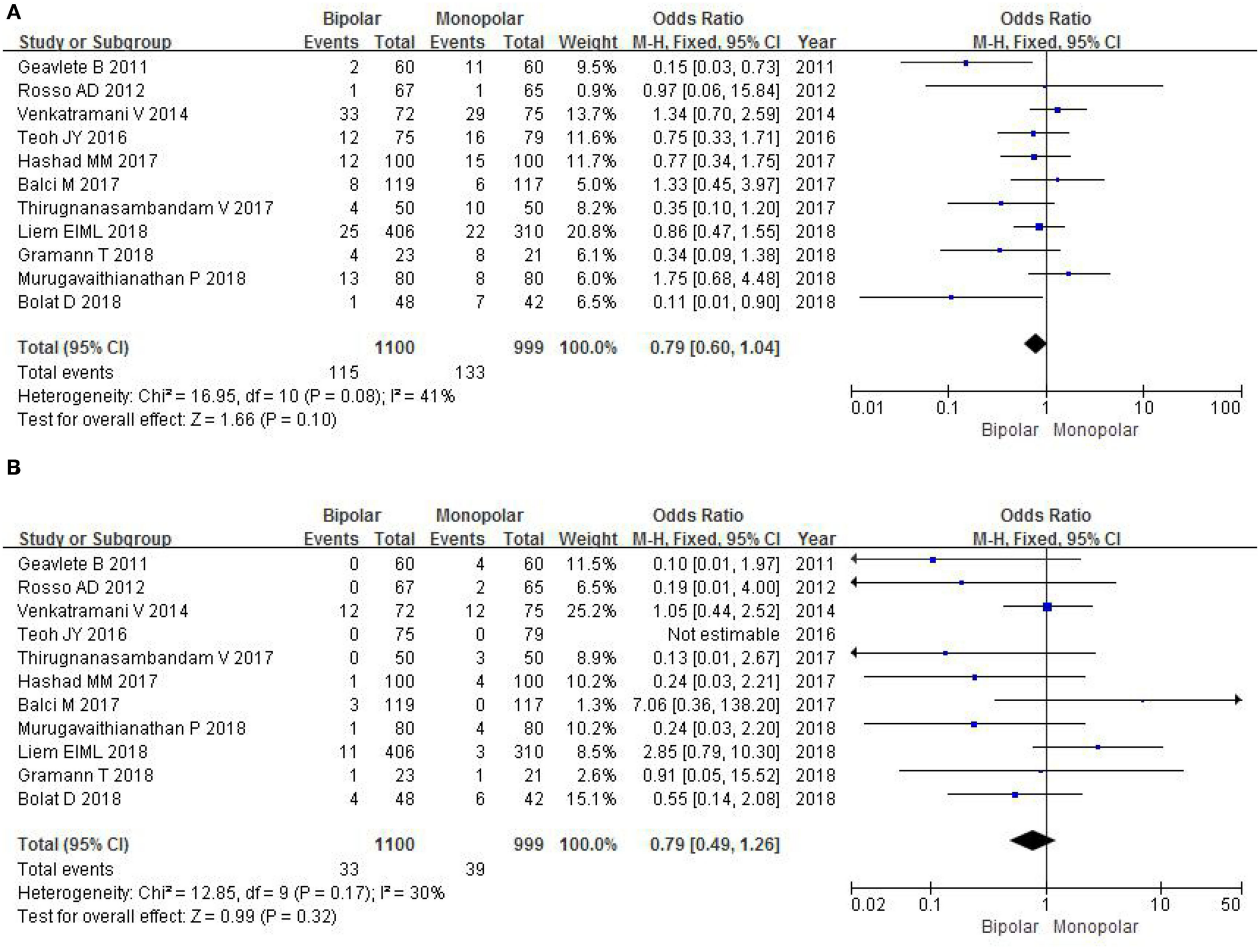
Forest plots showing numbers in **(A)** obturator reflex; **(B)** bladder perforation. M-H, Mantel–Haenszel; CI, confidence interval; df, degrees of freedom.

Eleven RCTs with a sample of 2,099 patients evaluated the severity of obturator reflex. The study showed that there is no statistical significance between b-TURB and m-TURB in the incidence of obturator reflex (OR 0.79, 95% CI 0.60–1.04, *P* = 0.10; Figure 7A).

#### Bladder Perforation

Eleven RCTs accessed the severity of bladder perforation with a sample size of 2,099 patients. The OR was 0.79, and 95% CI was 0.49–1.26 (*P* = 0.32; Figure 7B). This result suggested that b-TURB did not show a significant difference in the incidence of bladder perforation compared with m-TURB.

#### Postoperative Blood Transfusion

**Figure 8:**
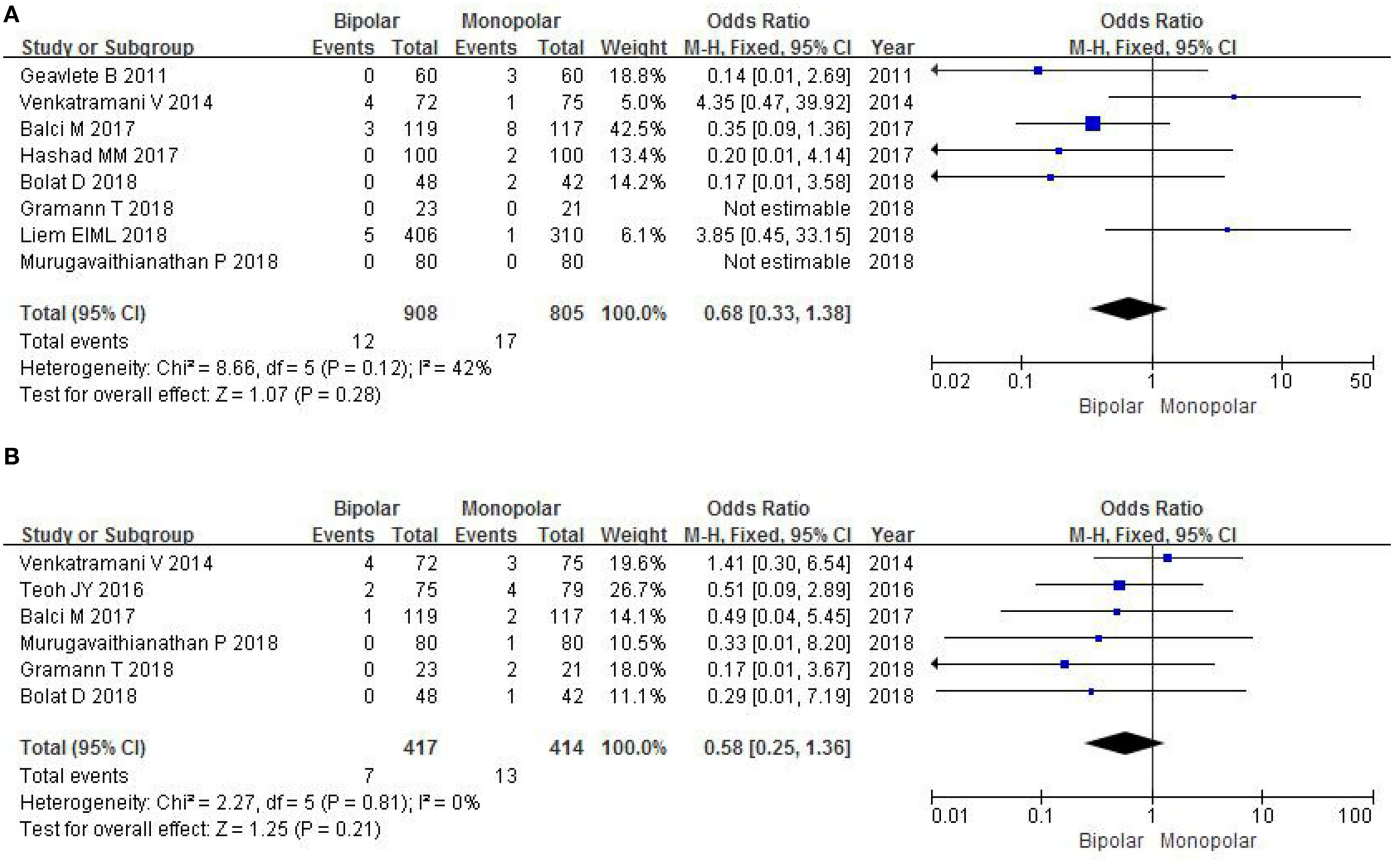
Forest plots showing numbers in **(A)** postoperative blood transfusion; **(B)** clot retention. M-H, Mantel–Haenszel; CI, confidence interval; df, degrees of freedom.

Eight RCTs with a sample of 1,713 patients analyzed the number of postoperative blood transfusion. A fixed-effects model did not show a statistical significance between b-TURB and m-TURB in the occurrence rate of blood transfusion after operation (OR 0.68, 95% CI 0.33–1.38, *P* = 0.28; Figure 8A).

#### Clot Retention

Six RCTs with a sample of 831 patients analyzed the number of clot retention. A fixed-effects model showed that there was not statistical significance between b-TURB and m-TURB in the occurrence rate of clot retention after operation (OR 0.58, 95% CI 0.25–1.36, *P* = 0.21; Figure 8B).

#### Muscle Tissue Sampling

**Figure 9:**
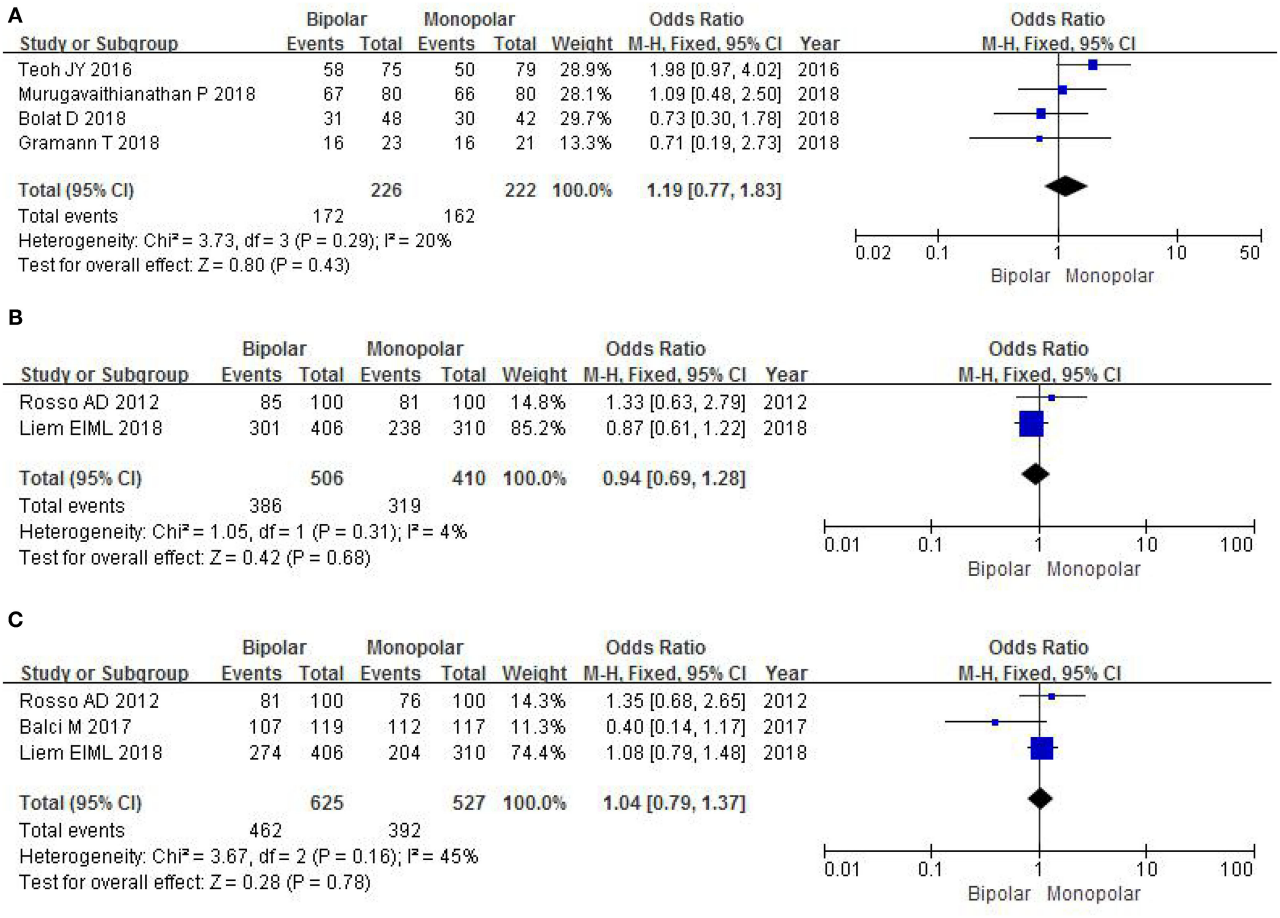
Forest plots showing numbers in **(A)** muscle tissue sampling; **(B)** recurrence-free survival at 6 months; **(C)** recurrence-free survival at 12 months. M-H, Mantel–Haenszel; CI, confidence interval; df, degrees of freedom.

Four RCTs evaluated the number of muscle tissue sampling with a sample of 448 patients. The study found that there was no significant difference between b-TURB and m-TURB in muscle tissue sampling rates (OR 1.19, 95% CI 0.77–1.83, *P* = 0.43; Figure 9A).

#### Recurrence-Free Survival at 6 Months

Two RCTs with a sample of 916 patients evaluated postoperative recurrence-free survival at 6 months. The study did not show a significant difference between b-TURB and m-TURB in recurrence-free survival at 6 months (OR 0.94, 95% CI 0.69–1.28, *P* = 0.68; Figure 9B).

#### Recurrence-Free Survival at 12 Months

Three RCTs evaluated postoperative recurrence-free survival at 12 months with a sample of 1,152 patients. A fixed-effects model showed that there was no significant difference between b-TURB and m-TURB in recurrence-free survival at 12 months (OR 1.04, 95% CI 0.79–1.37, *P* = 0.78; Figure 9C).

### Grading of Evidence

Evidence overview of primary and secondary outcomes of b-TURB vs. m-TURB is shown in Tables 3, 4. Grading of quality of evidence was downgraded to moderate because it was unclear whether the population undergoing bipolar or monopolar TUR was representative of the whole study population.

**Table 3:** Evidence overview of primary outcomes of bipolar vs. monopolar transurethral resection of bladder tumors.

**Outcome**	**No. of trials (evaluated)**	**Intervention, % (*n*/*N*) or mean**	**Control, % (*n*/*N*) or mean**	**Statistical model**	**Results and magnitude of effect (95% CI)**	**Strength of evidence**
Hemoglobin level	7 (1,038)	−0.54 points	−0.79 points	Random	Greater with monopolar TUR: MD −0.26 (−0.48 to −0.04)	Moderate^a^
Hematocrit level	3 (447)	−2.01 points	−3.03 points	Random	Greater with monopolar TUR: MD −1.16 (−2.06 to −0.26)	Low^a,b^
Sodium level	6 (865)	−1.13 points	−1.53 points	Random	Greater with monopolar TUR: MD −0.36 (−0.62 to −0.10)	Moderate^a^
Operation time	11 (2,099)	34.36 min, range 21.40–41.60	36.53 min, range 24.84–49.00	Random	Similar between groups: MD −1.73 (−6.38 to 2.92)	Moderate^a,c^
Catheterization time	5 (678)	1.58 day, range 0.87–2.50	1.98 day, range 0.98–3.50	Random	Similar between groups: MD −0.41 (−1.02 to 0.21)	Moderate^a,c^
Hospitalization time	8 (1,192)	2.12 day, range 1.30–3.50	2.57 day, range 1.50–4.50	Random	Greater with monopolar TUR: MD −0.44 (−0.82 to −0.07)	Moderate^a,b^

*CI, confidence interval; MD, mean difference; TUR, transurethral resection*.

*Downgraded based on the following*.

a *Risk of bias (moderate or high)*.

b *Imprecision*.

c *Unknown consistency or inconsistency*.

**Table 4:** Evidence overview of secondary outcomes of bipolar vs. monopolar transurethral resection of bladder tumors.

**Outcome**	**No. of trials (evaluated)**	**Intervention, % (*n*/*N*) or mean**	**Control, % (*n*/*N*) or mean**	**Statistical model**	**Results and magnitude of effect (95% CI)**	**Strength of evidence**
Obturator reflex	11 (2,099)	10 (115/1100)	13 (133/999)	Fixed	Similar between groups: OR 0.79 (−0.48 to −0.04)	Moderate^a,c^
Bladder perforation	11 (2,099)	3 (33/1100)	4 (39/999)	Fixed	Similar between groups: OR 0.79 (0.49–1.26)	Moderate^a,c^
Postoperative blood transfusion	8 (1,713)	1 (12/908)	2 (17/805)	Fixed	Similar between groups: OR 0.68 (0.33–1.38)	Moderate^a,c^
Clot retention	6 (831)	2 (7/417)	3 (13/414)	Fixed	Similar between groups: OR 0.58 (0.25–1.36)	Low^a,b^
Muscle tissue sampling	4 (448)	76 (172/226)	73 (162/222)	Fixed	Similar between groups: OR 1.19 (0.77–1.83)	Moderate^a^
Recurrence-free survival at 6-months	2 (916)	76 (386/506)	78 (319/410)	Fixed	Similar between groups: OR 0.94 (0.69–1.28)	Low^a,b^
Recurrence-free survival at 12-months	3 (1,152)	74 (462/625)	74 (392/527)	Fixed	Similar between groups: OR 1.04 (0.79–1.37)	Low^a,b^
TUR syndrome	6 (1,152)	0 (0/444)	1 (2/443)	Studies not pooled	Studies not pooled	Insufficient^b^
Postoperative severe cautery artifact	4 (1,152)	? (?/306)	? (?/299)	Studies not pooled	Studies not pooled	Insufficient^b^

*CI, confidence interval; MD, mean difference; TUR, transurethral resection*.

*Downgraded based on the following*.

a *Risk of bias (moderate or high)*.

b *Imprecision*.

c *Unknown consistency or inconsistency*.

## Discussion

In the past years, m-TURB has still been thought of as a standard surgery for NMIBC, which was frequently associated with the more postoperative complications (27, 28). Bipolar energy, as an alternative to TUR, has developed rapidly in recent years (29). The main superiority of b-TURB is the ability to cut cleanly, reduce tissue burning, and provide clear vision due to the use of saline (8).

This meta-analysis including 11 RCTs with a sample of 2,099 participants aimed to compare bipolar vs. monopolar energy for TURB. The results found that m-TURB had a greater decrease in postoperative hemoglobin level, hematocrit level, and sodium level compared with b-TURB. Besides, patients using b-TURB spent relatively less time in the hospital than those with m-TURB. However, with respect to intraoperative complications, mainly including obturator reflex, bladder perforation, postoperative blood transfusion, and clot retention, there were no significant differences between the two techniques. Meanwhile b-TURB did not show a relative advantage than m-TURB in both intraoperative operation time and postoperative catheterization time.

B-TURB is a novel technology wherein the positive pole and the negative pole are on the same axis and separated from each other through a ceramic connector (30). No reflux current improved hemostasis and minimized blood loss during resection (31). The average coagulation depth of b-TURB is greater than the maximum micro-vessel diameter, and its hemostatic ability may be better than m-TURB (32). B-TURB can coagulate venous bleeding, providing a clearer view of the surgery compared with m-TURB, reducing the time of surgery and the incidence of early complications (32).

M-TURB is flushed with a mannitol solution that may cause TUR syndrome, which is one of the most important complications for patients; in contrast, b-TURB with perfusion of saline during the operational process can avoid the occurrence of TUR syndrome (29, 33). In the analysis, six RCTs (18–21, 23, 26) had a relevant statistic on the number of patients with TUR syndrome after operation. Among them, five RCTs (19–21, 23, 26) did not report the occurrence of this complication, and only one RCT (18) reported two patients with TUR syndrome in the m-TURB group. This result showed that there appears to be no obvious difference between b-TURB and m-TURB in the occurrence rate of TUR syndrome after operation.

In terms of postoperative severe cautery artifact, four RCTs (17, 18, 21, 23) contained some inconsistent statistics about the number of patients, so the present study cannot make a systematic analysis on this point. Venkatramani et al. (18) demonstrated that the percent of severe cautery artifact was significantly lower in the b-TURB than in the m-TURB (25 vs. 46.7%, *P* = 0.0096). The other three RCTs (17, 21, 23) showed that there was no statistical significance between b-TURB and m-TURB in the number of patients with severe cautery artifact.

For the secondary outcomes, including muscle tissue sampling and recurrence-free survival at 6 and 12 months, the study did not show a significant difference between b-TURB and m-TURB, which indicated that the difference in technique did not affect the depth of tumor cutting and the postoperative recurrence rate of bladder tumors. Del Rosso et al. (17) revealed that the median time until bladder recurrence after initial TUR was 12.4 and 11.9 months for b-TURB and m-TURB, respectively. The 2-year recurrence-free survival rates were, respectively, 67 and 60%, and the Kaplan–Meier curve showed no significant difference (*P* = 0.70) in the overall recurrence-free survival rate between b-TURB and m-TURB (17). It is noteworthy that the results of the above study may be affected by postoperative management such as early instillation with saline, intravesical chemotherapy, and cystoscopic evaluation (34).

There has been increasing interest in *en bloc* resection of bladder tumor (ERB) as an ontologically non-inferior alternative to TURB with fewer complications and better histology specimens recently (35). Teoh et al. did a systematic review and meta-analysis for a total of 10 RCTs, which showed that ERB had a shorter irrigation time and a lower rate of bladder perforation than TURB, both with no significant differences in recurrences at 0–12, 13–24, or 25–36 months (36). This study mobilized the international urology community to develop a consensus statement on ERB using transparent and robust methods, and important outcomes for future ERB studies were also identified (36).

Recently, although three new meta-analyses have been published to directly confirm our results, there are many differences in the design and analysis of each study. Among them, two papers lack the analysis of the following indicators: decrease in postoperative hemoglobin level and sodium level, postoperative blood transfusion, clot retention, muscle tissue sampling, and recurrence-free survival at 6 and 12-months (37, 38). In the Tzelves's review, RCTs and observational study were included for qualitative and quantitative synthesis, which will reduce the evidence strength of the study (39). For the main evidence from RCTs, we rated our confidence in the estimates of effect for the outcome as strength of evidence (SOE) as high, moderate, low, or insufficient. Obviously, Xie et al. (38) found that b-TURB has no significant advantages in efficacy and safety in NMIBC treatment compared with that in m-TURB. Thus, b-TURB could not completely replace m-TURB as a safer and more effective NMIBC treatment. However, our study found that the bipolar technique was more effective than the monopolar technique in minimizing intraoperative or postoperative bleeding with relatively small loss of hemoglobin and a shorter hospitalization time for NMIBC. In addition, if there was more than one publication resulting from the same patient cohort, the most recent publication would be used to analyze, and the study may not follow this principle and include studies from the same population.

Although those articles included in the study were all randomized controlled trials to reinforce the findings, we must acknowledge the limitations of this meta-analysis. Selection bias, subjective factors, and publication bias may also affect the final results of our study. One limitation of our findings is some variables, such as local culture, the skill and experience of the operating surgeon, efficacy of perioperative care, and tumor size and number. We note also that the quality of some studies is flawed, primarily in terms of study design, patient selection, blinding, and outcome data. Therefore, the results of a meta-analysis should be interpreted carefully.

## Conclusion

The b-TURB was more effective than m-TURB in minimizing bleeding with relatively small loss of hemoglobin and a shorter hospitalization time. For operation time and catheterization time, both techniques did not show statistical significance. B-TURB did not show significant differences in the incidence rate of obturator reflex, bladder perforation, postoperative blood transfusion, and clot retention compared with m-TURB. Besides, there were no significant differences in terms of muscle tissue sampling, and recurrence-free survival at 6 and 12 months.

## Data Availability Statement

The original contributions presented in the study are included in the article/supplementary material, further inquiries can be directed to the corresponding author/s.

## Author Contributions

MY and YZ conceptualized and designed the study and provided administrative support. XM, ZZ, and YC collected, assembled, analyzed, and interpreted the data. All authors provided the study materials or patients, and wrote and gave the final approval of the manuscript.

## Conflict of Interest

The authors declare that the research was conducted in the absence of any commercial or financial relationships that could be construed as a potential conflict of interest.
